# Mountain Air Everywhere

**Published:** 1874-04

**Authors:** 


HALL'S

### JOURNAL OF HEALTH

AND

MISCELLANY.

Vol. XXI.—APRIL, 1874.—No. IV.

### MOUNTAIN AIR EVERYWHERE.

The mountain has at last come to Mahomet, and the delicious health and life giving atmosphere of the White Mountains, of a beautiful summer morning, can be introduced into every parlor in New York City, and a million of money can be saved to its citizens during the summer by their remaining at home and getting the sea or mountain air for as many hours every day, in their own houses, as they would have in the purest atmosphere "over the hills and far away." All understand that the purest, best, and most healthful atmosphere is found among the mountains, at sea, or in the "piny woods," but it is not so generally known that the reason of such greater healthfulness is the greater amount of a single ingredient—the fraction of a part only in a city at mid-day, one or two parts at the sea shore in the morning, and four or five parts on the mountain top. Twenty parts or more of this can be introduced in any room and kept there for a whole day. This agent is OZONE, which may be called an electric oxygen, and which consumes every impurity, and will destroy everything except water, oxygen and platinum. Its effect is to burn up every ingredient of impurity in the atmosphere, and to supply, besides, an element of life, vigor and elasticity to both mind and body, to a still greater extent than the pure air of the country.

THE POISON IN THE AIR AND ITS REMEDIAL AGENT.

It is a very old experience that the open air exerts a wholesome influence on the sick organism, improves the complexion and strengthens the muscles; and, just as well, we perceive that a continued residence in closed rooms, even in the most luxurious apartments, will produce pallor of the face, general weakness, diminution of the mental capacity, and that prison life will even totally undermine and destroy health, no matter under what form of disease.

We question, how is it possible that a body, apparently the same inside and outside of houses, could have such different, nay, even opposite effects? Different effects in our body can only be produced by different matter received into it, and we come to the conclusion that the air in and outside of our residences, although equally colorless, tasteless, and without smell, must be of different component parts. We know, since the last century, that air is a mixture of oxygen and nitrogen, and that breathing simply is a hunger for oxygen, and that a grown person needs for his existence more than 1,000 quarts (48 cubic feet) of oxygen every twenty-four hours. In view of this fact, it was the generally accepted opinion that there was more oxygen in the open air and less nitrogen, and that the air in closed rooms contained less oxygen and more nitrogen. But the examination of the air in the highest mountains, the deepest valleys, in forests, in closed rooms, crowded theatres and churches, gave an entirely different result. It turned out that the proportion of oxygen and nitrogen were everywhere the same under all conditions, in all places and at all times—the air was found to contain $\frac{1}{5}$ oxygen and $\frac{4}{5}$ nitrogen.

Science for a long time could not explain those mysterious different effects of the air, and it is only lately that the cause of this difference was clearly demonstrated. The air contains small organisms, not visible to the eye, which are called vibrios. Pasteur, the French *savan*, proved that those vibrios were the only excitors of putrefaction. Vibrios are capable of an immeasurably large increase, and they would soon fill the air in such quantities as to suffocate both animals and plants, if nature did not produce a gas, or a kind of air continually, which confines the increase of vibrios, and which allows their presence only to a certain extent.

The haunts of the vibrios are the ground and its surface, and higher up in the air this gas is generated, which is the deadly enemy to vibrios, destroying them wherever they come in contact. But of what nature is this gas? It is ozone, a negatively electric oxygen. Common oxygen represents $\frac{1}{5}$ of the whole atmosphere, but ozone is only present in the open air in small quantities—.0001 to .001—because, in its fight with the vibrios, it is just as quickly destroyed as it is generated. Ozone is generated in larger quantities, during storms by lightning, from the green leaves of plants and from the evaporations of solutions containing salt. Ozone is, so to say, oxygen loaded with electric power. It compares with common oxygen like diamond with graphite or coal. Ozone being more dense, and $1\frac{1}{2}$ times more heavy than oxygen, descends, therefore, to the earth from which the vibrios ascend. It has also a strong (phosphoric) smell, and has the important property to combine with all bodies except gold, platinum and water. This property is its great weapon in the destruction of the vibrios.

The open air is the space in which ozone continually rules, and vibrios have the supremacy in our houses, as ozone in them is very seldom or never recognized.

Every breath we take inside of our houses begins to poison and to weaken our body, every breath we take in the free air begins to neutralize poisons and to give strength.

